# Climatic, Geographic and Operational Determinants of Trihalomethanes (THMs) in Drinking Water Systems

**DOI:** 10.1038/srep35027

**Published:** 2016-10-20

**Authors:** Maria Valdivia-Garcia, Paul Weir, Zoe Frogbrook, David W. Graham, David Werner

**Affiliations:** 1 School of Civil Engineering and Geosciences, Newcastle University, Newcastle upon Tyne, United Kingdom; 2 Scottish Water, Castle House, Dunfermline, Edinburgh, United Kingdom

## Abstract

Trihalomethanes (THMs) are conditionally carcinogenic compounds formed during chlorine disinfection in water treatment processes around the world. THMs occur especially when source waters are subject to marine influences, high and-or regular precipitation, and elevated levels of organic matter. THMs formation is then rooted in geographic, operational and climatic factors, the relative importance of which can only be derived from large datasets and may change in the future. Ninety three full-scale Scottish water treatment plants (WTPs) were assessed from Jan 2011 to Jan 2013 to identify factors that promote THMs formation. Correlation analysis showed that ambient temperature was the primary THMs formation predictor in potable water (r^2^ = 0.66, *p* < 0.05) and water distribution systems (r^2^ = 0.43, *p* = 0.04), while dissolved organic carbon (r^2^ = 0.55, *p* < 0.001) and chloride (indicating marine influence; r^2^ = 0.41, *p* < 0.001) also affected THMs formation. GIS mapping of median THMs levels indicated brominated THMs were most prevalent in coastal areas and on islands. This real-world dataset confirms both geographic and climatic factors are key to THMs formation. If ambient temperatures increase, THMs control will become more challenging, substantiating concerns about the impact of global warming on water quality.

Chlorine disinfection is the most common and inexpensive way of eliminating pathogens from water to avoid serious water borne diseases such as diarrhoea, typhoid and cholera. However, chlorine-based disinfectants produce undesirable disinfection by-products (DBPs) such as trihalomethanes (THMs): chloroform (CHCl_3_), bromodichloromethane (CHBrCl_2_), dibromochloromethane (CHClBr_2_) and bromoform (CHBr_3_), which are closely monitored due to their suspected adverse human health effects^1^,^2^. In this context, the World Health Organisation (WHO) establishes the international standards for drinking water and states that primary consideration should be given to ensure that water disinfection is never compromised, but has nonetheless defined guidance values for individual THM compounds. These values are based on health criteria such as 10^−5^ excess lifetime cancer risks, and tolerable daily intakes for threshold effects, and are 300 μg/L for chloroform, 100 μg/L for dibromochloromethane and bromoform, and 60 μg/L for bromodichloromethane^3^. For total THMs, the WHO recommends a fractionation approach to account for additive toxicity. Based on WHO guidelines and the opinion of the European Commission’s Scientific Advisory Committee, the 1998 European Union Drinking Water Directive defines 100 μg/L as the allowable maximum concentration of total THMs (comprising chloroform, bromodichloromethane, dibromochloromethane and bromoform) in drinking water^4^. The US EPA regulates THMs at a maximum allowable annual average level of 80 μg/L. The US EPA additionally regulates another group of DBPs called haloacetic acids (HAAs) at a maximum permissible level of 60 μg/L^5^ for five compounds (HAA5). Other, unregulated DBPs may be formed in water disinfection, but it is generally accepted that measures taken to reduce organic THM precursors through multistep water treatment before disinfection should also reduce the formation of other DBPs^6^,^7^.

THMs form through the reaction between chlorine disinfectants and a pool of natural organic matter (NOM) present in water, often quantified as dissolved organic carbon (DOC). Therefore, in locations where the raw water source is rich in NOM (i.e., DBP precursors), minimization of THMs formation during water treatment and distribution can be a challenge. Scotland is a case in point. The total volume of water abstracted for drinking water is 1600 ML/d, with 87% coming from surface water sources (lochs, reservoirs, rivers and springs) and 13% from groundwater (http://www.gov.scot/Publications/2004/04/19262/36053). Scotland has 75% of the peatland in the UK, is surrounded by marine waters, and a great percentage of its territory is islands^8^. Scottish geography provides raw waters of diverse chemical composition. For example in the Highlands, granitic parent soil and scarce grassland makes soils organic-rich and soft, whereas in the lowlands, fresh water bodies are often alkaline and higher in nutrients originating from forested peatland and vegetative decay. The diversity of organic compounds present in surface water is vast and dependent on the sources where they originate^9^,^10^,^11^. An important characteristic of surface waters in Scotland is discolouration. Brown to yellow colours are common in rivers and lochs, which is attributed to soils rich in humic and fulvic acids. Similarly, phenolic compounds produced by vegetation decay are also released by rainfall and runoff events into surface waters^12^,^13^.

Within this context, climate change projections have suggested Scottish temperatures will increase and precipitation will become more variable in the future, increasing microbial and chemical reaction rates, potentially altering DOC levels in surface waters, and bringing new challenges in drinking water treatment^8^,^14^,^15^,^16^. Hydrological changes such as water table levels fluctuations, produced by rainfall or drought can increase or decrease in situ DOC levels. When water table levels drop during summer due to natural water evaporation, microbial activity increases producing higher levels of DOC in soil layers^17^. Rainfall may then contribute to the release of larger quantities of carbon compounds from organic rich soils, whereas this is less likely from granite, mudstone and sandstone soils, which will release mainly inorganic compounds^17^,^18^. Organic and organo-mineral soils will release compounds with lower molecular weight as a result of microbial degradation^19^. Further, freshwater bodies near coastal areas will be affected by easterly or westerly winds, with impacted rainwater adding marine salts, altering freshwater composition^18^. Dissolved halides (bromide, chloride and iodide) from marine sources have been previously correlated with THMs formation^20^,^21^,^22^, creating particular problems in water treatment on marine islands.

The array of possible causes of THMs formation is diverse. Therefore, this work was performed to identify “best predictors” of THMs levels in final potable water and distribution networks, and determine how THM formation rates might change in the future. Specifically, large regulatory monitoring datasets were assessed from 93 full-scale drinking WTPs in Scotland to distinguish among geographical, large-scale anthropogenic and operational factors on THMs formation on a country-scale. The ultimate goal here was to quantify relationships between detected THMs levels, and the seasonality and diversity of DOC across the region, and translate those observations to a deeper understanding how climate change will impact THMs formation and treated water quality in the future.

## Results

### Spatial analysis

**Figure 1:**
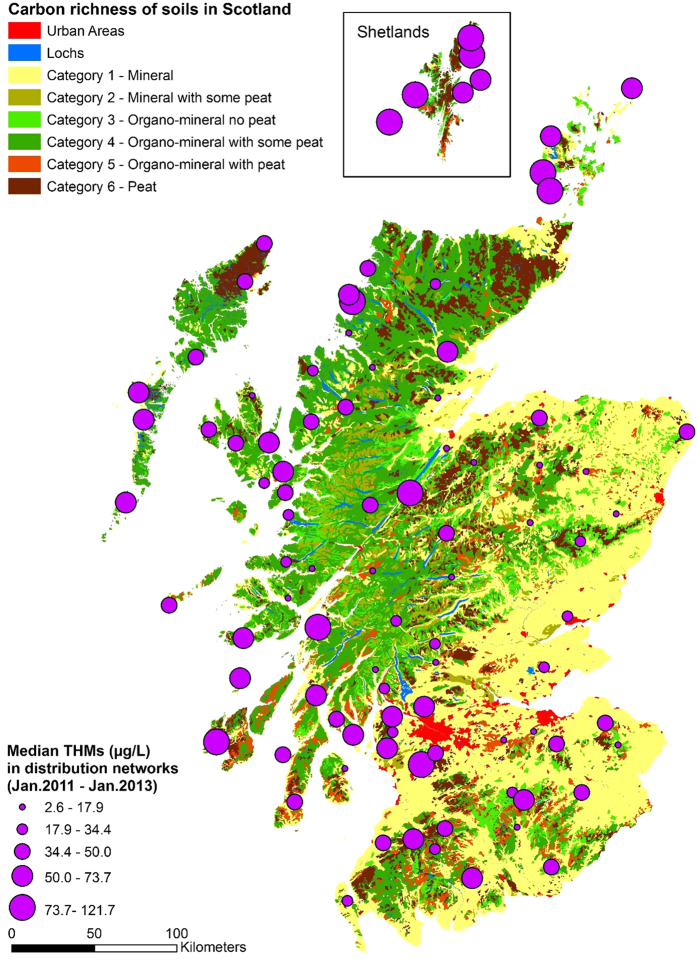
**Spatial distribution of median total THMs on 93 drinking water plants around Scotland on a carbon richness soil layer (Jan. 2011–Jan. 2013) (Obtained using** **http://www.esri.com/news/arcnews/spring12articles/introducing-arcgis-101.html****; version ArcMap 10.1**).

Soils across Scotland are highly varied, ranging from organic carbon-rich soils that form peatlands, bogs and marshes that predominate in the west of Scotland, to brown earths and humous iron podzols that include agricultural land often more located in eastern Scotland. Using these data, plots describing median THMs concentrations in water distribution networks associated with the 93 WTPs were overlaid onto a map of soil types across Scotland (Fig. 1).

**Figure 2:**
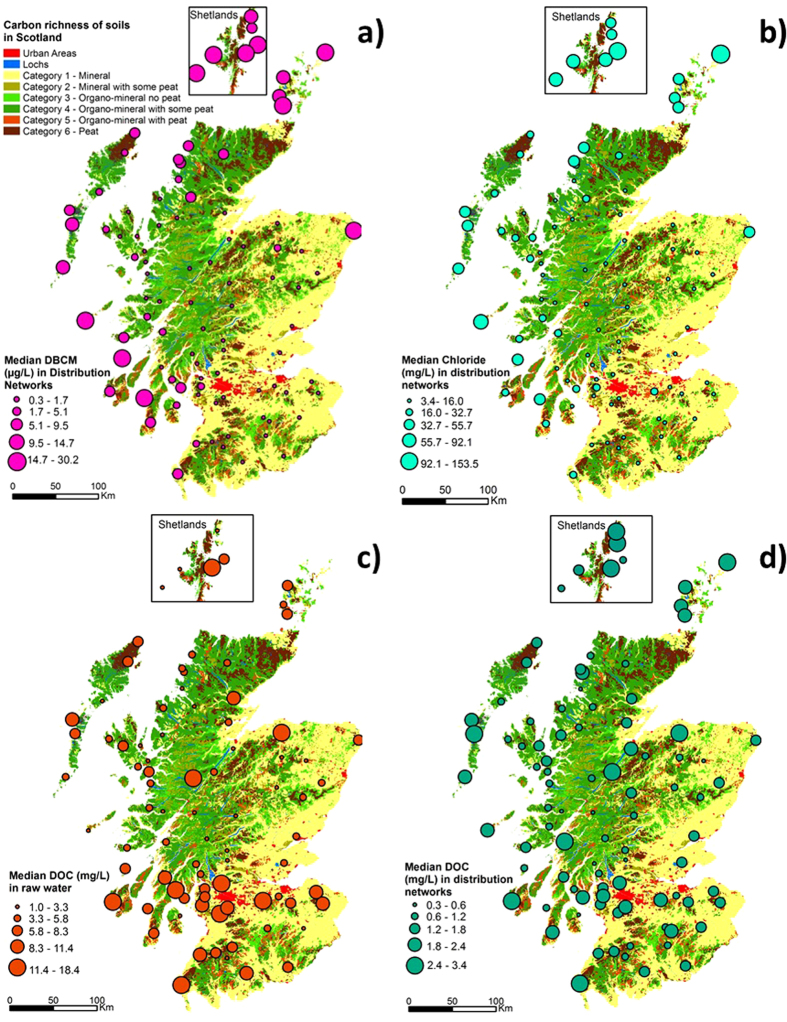
Median values for (**a**) Dibromochloromethane (DBCM), (**b**) chloride, (**c**) DOC (raw water) and (**d**) DOC (distribution networks) at 93 drinking water treatment plants in Scotland (Jan. 2011–Jan. 2013). (Obtained using http://www.esri.com/news/arcnews/spring12articles/introducing-arcgis-101.html; version ArcMap 10.1).

The largest median THMs levels were most often found in coastal proximities and in the west (Fig. 1) where peat is abundant and precipitation is high (Fig. S1 can be found in Supplementary information), whereas lower THMs levels were found in the Eastern Mainland. The most obvious spatial pattern was for brominated THMs compounds such dibromochloromethane (Fig. 2a), which were primarily found on islands and associated with WTPs sites near the coast, which implies a strong influence of marine halides on associated THMs formation in distribution systems. Specifically, the spatial distribution of dibromochloromethane shows a clear link between brominated THMs and high levels of marine chloride found at coastal sites (Fig. 2a,b). The presence of rich organic soils and peatland with halides from marine influence provide a perfect precursor combination for THMs formation.

Median DOC values for each drinking WTP were also overlaid on the soil type distribution map, allowing visualization of spatial trends between soil types, DOC in raw water and DOC in distribution networks (Fig. 2c,d), but the relationships are not as clear as between coastal proximity, chloride levels and brominated THMs formation.

### Temporal trends

**Figure 3:**
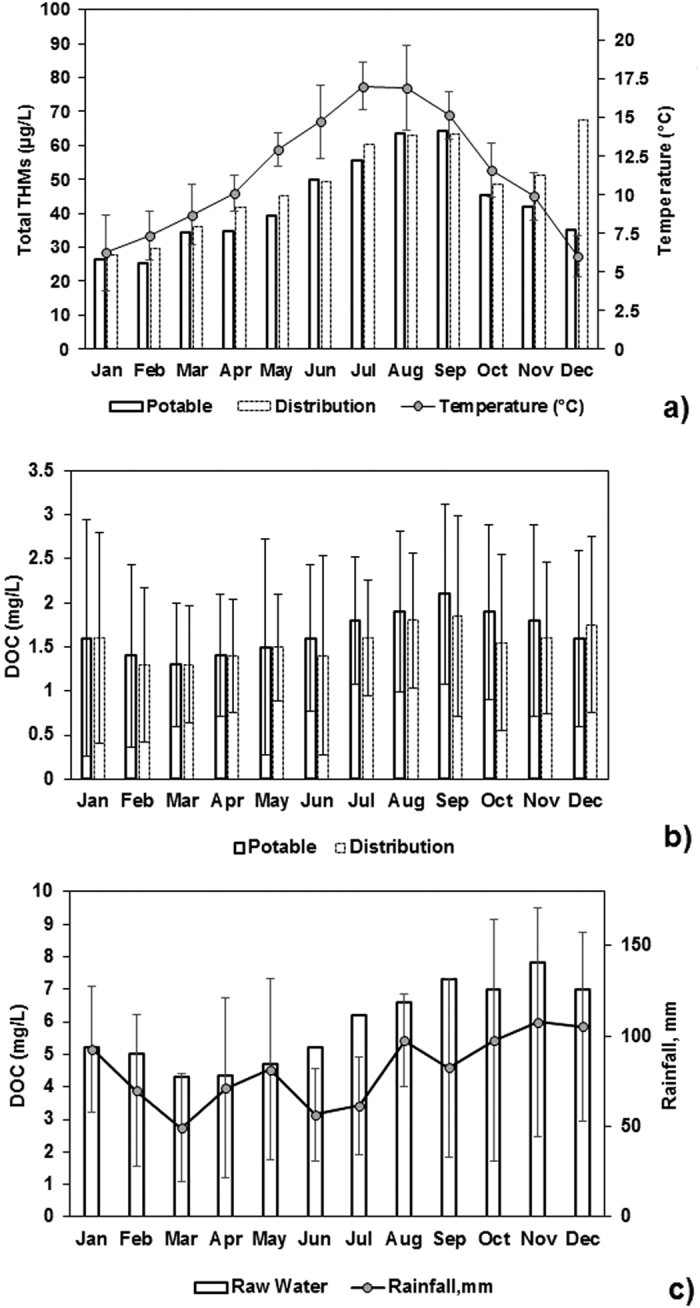
Bar plots with standard errors for: (**a**) median total THMs in potable and distribution samples with ambient temperature (error bars not shown for better visibility but typical ranges appear in Table 1), (**b**) median DOC in potable and distribution samples, (**c**) median DOC in raw water with rainfall levels (Jan. 2011 to Jan. 2013).

Mean DOC and THMs levels across potable water and distribution networks displayed similar seasonal changes with ambient temperature and local rainfall (Fig. 3). Based on Meteorological Station data from across Scotland, the highest recorded monthly temperatures were in July, 2011 and August, 2012 with 16.5 ± s.e. 0.69 °C and 17.4 ± s.e. 0.56 °C, respectively. In terms of rainfall, highest mean levels were found in December 2011 and 2012 with 189.8 ± s.e. 39.3 mm and 145.1 ± s.e. 16.2 mm, respectively.

For example, the strongest seasonal influence on total THMs in potable water is ambient temperature (Fig. 3a), although the seasonal temperature maxima in July precedes the median total THMs maxima in September (i.e., by about 2 months). This can be explained by DOC levels in potable water, which also influence THMs levels, but peak in September (Fig. 3b), suggesting higher temperatures associated with elevated DOC levels results in higher THMs levels. In contrast, the combination of lower ambient temperatures from Jan-April and lower potable water DOC explain lower total THMs levels observed in distribution networks in the first third of the calendar year. Raw water DOC (Fig. 3c) generally follows seasonal rainfall levels, with higher median values being recorded in the second half of the year. As the water table drops during summer months the microbial activity increases, elevating DOC production which is flushed out of the system by rainfall events and continues to do so until the water table increases again in the winter^17^,^23^.

Ambient temperature plays an important role in chemical reaction kinetics and disinfection practice. Chlorine consumption in distribution networks is accelerated by high temperatures, and during summer months excess chlorine is sometimes added to maintain minimum residual levels. This additional chlorine dosing will result in more THMs formation, which may partially explain the observed seasonal trends. However, temperature dependency of THMs formation and also decay in distribution networks is very complex^24^,^25^. For example, a temperature-dependant kinetic effect is seen in the marked increase of THMs in distribution networks relative to final potable water THMs in December. This is potentially because lower temperatures slow down the rates of THMs formation during primary disinfection, which then increase from reactions with residual chlorine during transport in the distribution systems. Temperature effects on THMs formation were less evident in WTPs using chloramines instead of chlorine as disinfectant residual, as will be discussed in more detail later. Regardless of the exact mechanism, the observed relationship with ambient temperature suggests that global warming may exacerbate the THMs formation potential in WTPs.

### Pearson’s bivariate correlation analysis for THMs and other water quality parameters in potable water and distribution systems

**Figure 4:**
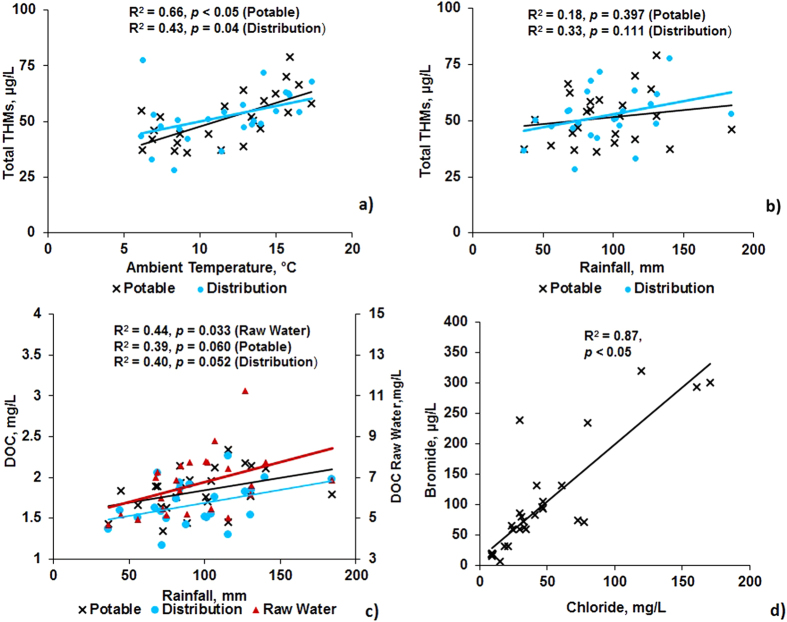
Correlation plots between monthly average concentrations (Jan. 2011–Jan. 2013) for (**a**) THMs and ambient temperature, (**b**) THMs and rainfall and (**c**) DOC and rainfall. (**d**) Correlations between bromide and chloride concentrations (Jan. 2011–Jan. 2013).

Pearson’s correlation analysis was applied to all measured data, and confirmed that ambient temperature, DOC and chloride were most influential to THMs formation across Scottish WTPs (Table 1). Verifying the findings of seasonal trends, monthly average temperatures showed a significant correlation with monthly average THMs in potable water (r^2^ = 0.66, *p* < 0.05) and distribution systems (r^2^ = 0.43 *p* = 0.04) (Fig. 4a). Greater correlation between THMs and ambient temperature in potable water than distribution networks can be due to the immediate reaction kinetics with chlorine. As contact time in distribution networks increases, the dependency of THMs formation in the networks relies on residual chlorine, DOC and temperature. Rainfall was not significantly correlated with THMs in potable water (r^2^ = 0.18, *p* = 0.397) (Fig. 4b) or distribution networks (r^2^ = 0.33, *p* = 0.256) (Fig. 4b). In the case of rainfall and DOC, a significant correlation was found for DOC in raw water (r^2^ = 0.44, *p* = 0.03), but not for potable water (r^2^ = 0.4, *p* = 0.06) or water in distribution networks (r^2^ = 0.4, *p* = 0.052) (Fig. 4c). Similarly, a local survey of water treatment plants in Beijing, China, reported weaker, but still positive, Pearson correlations between THMs with water temperature (r = 0.253, *p* < 0.05) and TOC (r = 0.176, *p* > 0.05), respectively^26^.

Unfortunately, bromide levels are not measured regularly, except at a few sites in the raw water (n = 30), but for the available measurements, chloride and bromide levels significantly correlate (r^2^ = 0.87, *p* < 0.05) (Fig. 4d). This observation is consistent with other studies where chloride and bromide also were correlated, such as in Australian surface waters where chloride is being used as a proxy for bromide in coastal areas^27^. This is relevant because, even at low concentrations, bromide promotes THMs formation as a first-order rate reaction^28^. Therefore, the presence of both halides during disinfection can increase reaction rates with DOC. One of the conclusions from our study is to routinely measure both ions as well as temperature in raw waters and distribution networks, especially in coastal areas, and also to consider alternative treatment technologies, including filtration (e.g. GAC) and-or ion exchange resins in such situations that can remove halides as THMs precursors prior to disinfection^22^.

Other weak, but statistically significant, correlations with total THMs in distribution networks, were observed with colour, conductivity and turbidity (Table 1). Due to the effectiveness of conventional treatment steps, such as coagulation, sand filtration, GAC, and membrane filtration, turbidity and colour are usually very low in most WTPs (80–95% removal). However, colour and DOC in raw water showed a strong correlation between each other (r^2^ = 0.78, *p* < 0.001, n = 994) and colour also correlated weakly but significantly with THMs in distribution samples (r^2^ = 0.25, *p* < 0.001, n = 954) and in potable water samples (r^2^ = 0.39, *p* < 0.001, n = 1187). We suspect the correlation between colour and DOC in raw water is primarily related to the presence of coloured phenolic compounds typically abundant in organic soils^29^,^30^.

### Similarities with haloacetic acids (HAAs) formation

No guidance parameter value has been set for HAAs in the European Union Drinking Water Directive, and these DBPs are therefore not as widely monitored as THMs, but available data suggests strong correlations of HAAs and THMs formation. Monthly average concentrations collected in 2014 for five haloacetic acids (HAA5) taken from distribution networks in one drinking water treatment plant in the West of Scotland also showed a strong and significant positive Pearson correlation with ambient temperature (r^2^ = 0.61, n = 12, *p* = 0.034) and DOC (r^2^ = 0.66, n = 12, *p* = 0.018). A strong and significant correlation was also found between THMs and HAAs monthly average values for this particular site during the same period (r^2^ = 0.68, n = 12, *p* = 0.015). In line with the findings of studies in China^31^, Canada^6^, England and Wales^32^,^33^, these correlations substantiate that the formation of THMs and other DBPs in water disinfection with chlorine have similar underlying causes.

### DOC removal efficiency

The mean DOC concentration in raw waters, potable waters and within distribution systems across Scotland were 6.6 ± s.e. 0.48 mg/L (n = 1233); 1.8 ± s.e. 0.02 mg/L (n = 2402) and 1.7 ± s.e. 0.02 mg/L (n = 1809), respectively (Table 2). Overall, DOC removal efficiencies across all WTPs was typically 65 to 75%, which was lower than colour (87%) and turbidity (77%) removal. In general, water treatment removes colour more effectively than DOC, leaving less coloured DOC residuals (typically lower molecular weight). This residual DOC fraction, often found in potable and distribution networks, might sustain microbial communities in water lines and potentially react in combination with chlorine and halides to form THMs.

### Treatment effects for ground water

Fourteen of the 93 WTPs in this study abstracted groundwater as their primary source water. Three of these sites used additional GAC filtration for treatment, whilst the other 11 used chlorination only because of good raw water quality in terms of turbidity (0.4 NTU) and colour (16 mg Pt/Co) which are values very close to drinking water. Average total THMs levels in distribution networks associated with these sites were significantly lower (mean 36.7 ± s.e. 1.7 μg/L) than sites with surface water sources (51.2 ± s.e. 0.5 μg/L; *t* = −8.1, *p* < 0.001). The mean total THMs levels from WTPs that use disinfection only in distribution networks (36.2 ± s.e. 3.6 μg/L) vs disinfection plus GAC units (38.9 ± s.e. 3.6 μg/L) were not significantly different (*t* = −0.52, *p* < 0.601). GAC is typically only used at sites with poorer quality raw water. However, data here indicate GAC has limited additional benefit to reducing THMs levels. It should be noted that inclusion of GAC filtration can improve THMs precursor removal^34^,^35^ but the specific adsorbent must be chosen carefully^22^. Further work needs to be done to better understand the choice of GAC utilized at these groundwater sites and their efficacy for THMs precursor removal.

### Treatment effects for surface water

Coagulation is one of the most common treatment methods employed to produce potable water in Scotland and is included in ~50% of the WTPs in this study. The common coagulant is aluminium sulphate supplemented with polyelectrolyte (0.1 mg/L polyacrylamide) and coupled pH adjustment to between 5.8 and 6.3 which is then raised to 8 as water leaves the WTPs to prevent network corrosion. In reviewing THMs levels from WTPs with coagulation, the lowest THMs values were observed at WTPs with coagulation followed by ultrafiltration which produced total THMs levels of 21.1 ± s.e. 3.1 μg/l (n = 40) and 16.0 ± s.e. 1.1 μg/L (n = 41) in potable and distribution water, respectively (Table 3).

Coagulation with dissolved air flotation (DAF) and rapid gravity sand filters (RGF) produced similar THMs levels to WTPs with coagulation and pressure filtration, but sites that combine coagulation with RGF and GAC tended to have much higher THMs levels across all potable water and distribution system samples. For example, two-sample t-tests showed that sites using coagulation with GAC had a significantly higher mean THMs in distribution samples at 83.3 ± s.e. 5.3 μg/L (*t* = −8.9, *p* < 0.001) compared with all other WTPs with coagulation (mean 48.8 ± s.e. 0.9 μg/L). Similar to groundwater sources, our results show THMs precursor removal is not necessarily substantially enhanced by an additional GAC treatment step which reinforces the requirement of further study around this area.

When evaluating WTPs with membrane filtration, sites with hollow fibre ultrafiltration (UF) membranes performed much better than other WTPs, producing the lowest THMs levels (Table 3), which may, however, be due to the low raw water DOC of the hollow fibre UF plants. Of membrane options, spiral UF had the highest THMs levels (51.3–55.9 μg/L) in potable and distribution water samples which relates to their higher molecular weight cut-off (MWCO). However, total THMs levels were not significantly different between membrane and coagulation plants (including GAC filtration) (*t* = 0.99, *p* = 0.33) which again will be influenced by inlet DOC loadings.

In the case of less common treatment options, coagulation with Inverness (up-flow) filters and ozone with GAC treatment and yielded significantly higher total THMs levels in distribution networks (100.0 ± s.e. 2.8 μg/L) than traditional coagulation or membrane WTPs (46.6 ± s.e. 0.82 μg/L) (*t* = 18.5, *p* < 0.001). Total THMs in potable water found in DynaSand^®^-based WTPs (45.0 ± s.e. 2.0 μg/L) were evaluated against all other sand filtration treatments (47.1 ± s.e. 0.8 μg/L) and no significant difference were found in potable (*t* = −0.98, *p* = 0.328) or distribution water samples (*t* = 0.67, *p* = 0.502). These results show that conventional coagulation and membrane filtration systems are generally better options than non-conventional treatment options in terms of THMs formation trends for Scottish drinking water systems.

### Chlorination versus chloramination plants; Potable water versus distribution water

Scottish Water uses both chlorination and chloramination for disinfection. Eleven of the 93 WTPs within the network use chloramine as the disinfectant, whereas the remaining WTPs use chlorination. Chlorination and chloramination sites do not differ significantly in terms of total THMs levels in their final potable water, but THMs levels are significantly higher in water distribution networks with chlorination (53.5 ± s.e. 0.88 μg/L, n = 1716) vs chloramination (28.7 ± s.e. 3.8 μg/L, n = 238) (*t* = 11.96, *p* < 0.001). These results are partially explained by the loss of free chlorine on the addition of ammonia at the WTPs in the production of chloramine and the need for extra chlorine doses within networks with chlorination systems (e.g., in service reservoirs), which ensure isolated households have acceptable chlorine residuals (0.2–0.3 to 1 mg/L as free chlorine). It should be noted that this practice is performed more often in summer months when higher temperatures can cause more rapid depletion of chlorine^36^. In the case of chloramination WTPs, the higher stability of mono- and di-chloramines results in lower rates of disinfectant decay, which causes longer lasting residuals, considerably lower THMs formation, and less need for additional disinfectant in the distribution networks. This statement is corroborated by a very important finding of this study which is that the relationship found between ambient temperature and total THMs differs according to the type of disinfection. The data was separated into two sets one containing sites that use chlorination and another using chloramination (Fig. S2 in supplementary information). For the chlorination dataset a strong and significant correlation was found between THMs and ambient temperature monthly average values (n = 24) in potable water (r^2^ = 0.71, *p* < 0.05), and also in distribution networks (r^2^ = 0.48, *p* < 0.05). However, no such correlation was found between these two variables in the chloramination dataset. The finding indicates that WTPs using chlorination will be most affected by changes in ambient temperature.

Overall, these above results indicate THMs formation control also must consider phenomena in the distribution networks. In fact, 79% of the WTP systems (73 of 93) had statistically significant higher total THMs (*t* = −2.4, *p* < 0.001) in their distribution networks (51 ± s.e. 0.8 μg/L) than in their treated potable water (48 ± s.e. 0.6 μg/L). This implies net THMs formation reactions continue outside of the WTP itself and managing such reactions in the distribution system is key to minimizing THMs levels at the tap.

### Multilinear regression models for individual THMs compounds

Chlorination-based WTP systems display much stronger multivariate regression correlations for total THMs levels (r^2^ = 0.76, *p* < 0.05) than chloramination systems (r^2^ = 0.37, *p* < 0.05) with the main predictors being ambient temperature, chloride and DOC in chlorination sites and chloride and DOC for sites that use chloramination. Relative to specific THMs compounds, chloroform and bromodichloromethane are most associated with ambient temperature, chloride and DOC, whereas bromoform was only correlated with temperature and chloride in chlorination systems (Table 4). THMs prediction models indicate for example that concentrations higher than the annual average for each of the predictors will yield higher THMs. These models will facilitate the interpretation of results at the treatment sites and help operators and managers to control the process by setting temperature-dependant targets for residual DOC and halide concentrations in order to minimize THMs formation. We believe the negative correlation of chloroform with chloride is due to the preferential formation of brominated THMs from waters with high halide levels. Bromoform formation was often below 0.5 μg/L, which appeared to skew regression analysis, therefore below-detection limit bromoform data (0.3 μg/L) were not included in the regression analysis for the chlorination dataset. Elimination of such low values was not performed for chloramination sites due to small number of data entries and hence no correlation could be established for bromoform data (Table 4). It is then of great importance to identify bromide concentrations at the raw water and in the distribution networks in the future and thus to produce an improved prediction model for THMs formation.

Previously THMs studies also have used other predictors, including pH, UV, fluorescence, and C/N ratios, which can provide useful information on characteristics of THMs precursors^36^. However, this current investigation relied on monitoring data typically available to water utilities (a pragmatic approach), and we found that chloride and DOC, consistently predicted THMs levels, which we suspect is valuable to water companies for THMs management. However, ambient temperature data was incorporated into the multilinear regression model due to the evidence of seasonal changes affecting levels of THMs in chlorination plants which was corroborated with positive and strong associations. Such findings are very important because they bring into the attention that not just DOC and halide residuals at the disinfection point of water treatment are causing THMs formation, but external factors such as ambient temperature are also influential. Thus, rising temperatures caused by global warming will have an immediate effect on THMs formation and the economics of water treatment in the future.

## Discussion

THMs are conditionally carcinogenic compounds that are formed during chlorine disinfection. THM formation has been known for many years^37^, but most studies on THMs have been based upon local or laboratory assessments, which limits the scope of bigger picture predictions based on multiple real-world observations. For example, it is suspected water quality may decline as climate changes^38^, but it is very tenuous to make specific predictions without stronger and more extensive field data that confirm speculation. This is especially critical to the water industry, which must make major infrastructural decisions about future water systems and there is uncertainty about the climate within which they will operate.

Within this context, we assembled an extensive database that contained operating data from 93 full-scale WTP systems, including 46,999 data entries from across Scotland. To our knowledge, this is one of the largest assessments ever performed on water systems, especially related to THM formation as a function of geographic, operational, and climatic factors. Although the sampling frequency varied between WTPs and the data for some parameters were less complete than others, the dataset is still extensive and allows statistical comparisons among factors that impact THMs at a rigorous level. Overall, data show that DOC, which varies by location and regional weather (e.g., precipitation), chloride and especially ambient temperature conditions all significantly relate to THMs formed during water treatment across the Scottish network. The importance of such factors to THM formation has been observed previously^22^,^34^,^39^, but here we show such factors are manifestly important at a country-scale, which becomes very significant when one considers the possible impacts of climate change on the water industry.

Scottish data specifically show that warming temperatures and-or more variable precipitation will very likely change or exacerbate THM formation potential in regional WTPs. However, such observations have global implications, especially in countries that use regularly chlorination in water treatment, such as the United Kingdom or United States. For example, we observe much higher THMs levels in potable water systems with higher seasonal temperatures, which we suspect is related to accelerated formation kinetics and also altered DOC release from organic rich soils. If one considers projected increases in temperature of 2–3 °C within the next 40 years^40^ (which is within the Scottish temperature range), treatment adaptations, such as moving away from chlorination or applying enhanced DOC removal processes, may be needed to reduce impact of global warming on THMs formation and its possible health consequences. Although this has considerable operational implications to companies, we provide here a template for addressing this prospective problem, including implications of catchment management, different treatment options and infrastructural upgrades, which we hope will assist water companies with similar decisions around the world.

## Methods

### Sampling Methodology

Exploratory statistical analysis, multi-linear regression and data mining was applied to water quality parameters measured in Scottish Water Laboratories at different sampling points across their water network (i.e., raw water is surface water at the inlet of each WTP, final potable water refers to disinfected water at the treatment site; and distribution water refers to potable water samples taken at randomised customer taps) between January 2011 to January 2013. All monitored quality data were archived and then drawn from Scottish Water’s Laboratory Information Management System (LIMS). A summary of the data used in the statistical analyses appears in Table 2. As background, Scotland is divided into 16 geographical regions. The number of sites used in the analysis varied among regions, being allocated in a stratified manner to make resulting analyses representative. One-third (n = 93) of the total number of drinking water treatment sites (n = 270) was used to make the analysis workable. The actual number of sites per region is given by *n*_*i*_ (Table S1 in supplementary information).

Water samples were collected and analysed following a scheduled sampling programme and certified analytical protocols approved by the Drinking Water Quality Regulator (DWQR) for Scotland and the United Kingdom Accreditation Service (UKAS). THMs were measured using a modified in house method based on EPA Method 524.2 for purgeable organic compounds in water by capillary column gas chromatography mass spectrometry^41^,^42^. Soil data, used to describe background soil type and horizon data across Scotland, were provided by the James Hutton Institute (Aberdeen, Scotland). Rainfall and temperature data were collected from nine Meteorological Stations located across Scotland (Paisley, Dunstaffanage, Tiree, Stornoway, Lerwick, Wick, Nairn, Braemar and Leuchars), including data from January 2011 to January 2013 (historical data available from http://www.metoffice.gov.uk). Using these data, average and standard deviations for monthly rainfall and temperature were calculated. Larger WTPs in Scotland’s main cities have more wider-scoped sampling strategies than rural locations, which meant available data density varied from WTP to WTP across the country.

### Exploratory Statistics

The median values for quality variables in raw water, final potable water and distribution networks sampling points at the 93 WTPs were plotted using ArcMap 10.1 (ArcGIS, Environmental Systems Research Institute, CA, 2011) over a soil type layer based on the carbon richness grouped in six categories^43^. A data set that included median values for quality parameters measured at the 93 WTPs from January 2011 to January 2013 (at noted sampling points) was used to compare and visualize spatial distributions. Analysis of means and errors were calculated using Minitab 17 (Leadtools Technologies Inc, version 17.1.0, 2014) and reported with 95% confidence limits by showing plus minus standard errors of the mean.

### Correlations and multilinear regressions

Pearson correlations and multilinear regressions were calculated using Matlab R2015a (MathWorks, version 8.5, 2015). Bivariate correlations between measured variables in raw water, final potable water and distribution networks were performed using Minitab 17. Correlation analysis was also performed between bromide data measured from raw water and chloride in distribution network samples with a maximum threshold of three days between sampling dates. Finally, comprehensive multilinear regressions were performed using two data sub-sets from the original database that did not contain missing values: data from WTPs that used chlorine disinfection (n = 502) and WTPs that used chloramination (n = 65). In multilinear regressions for individual and total THMs (dependent variables), using a robust linear fit function (linfit, RobustOpts), only the predictors with high p-value were retained. The robust method option was chosen because it is less influenced by outliers than conventional least-square fit and transformation analysis, especially for non-normally distributed data. Annual average values were subtracted from correlation data to obtain a multilinear regression intercept corresponding to a representative THM concentration.

### Data Availability

The study brought together existing data obtained upon request and subject to licence restrictions from a number of different sources. Full details of data available in the documentation at: http://dx.doi.org/10.17634/120242-1.

## Additional Information

**How to cite this article**: Valdivia-Garcia, M. *et al*. Climatic, Geographic and Operational Determinants of Trihalomethanes (THMs) in Drinking Water Systems. *Sci. Rep.* **6**, 35027; doi: 10.1038/srep35027 (2016).

## Supplementary Material

Supplementary Information

## Figures and Tables

**Table 1:** Bivariate Pearson correlations between quality variables in potable and distribution water samples with THMs.

Quality Variables	Final potable water			Distribution networks		
	r^2^	p	n	r^2^	p	n
*Alkalinity (mg HCO*_*3*_^−^/*L*)	**0.17**	0.06	125	**n.a**	n.a	n.a
*Ammonium (mg NH*_*4*_^+^/*L*)	**−0.39**	0.00	114	**0.01**	0.87	924
*Colour (mg Pt*/*Co*)	**0.39**	0.00	1187	**0.25**	0.00	954
*pH*	**0.02**	0.61	1187	**0.00**	0.90	939
*DOC (mg C*/*L*)	**0.47**	0.00	2320	**0.55**	0.00	1740
*Turbidity (NTU*)	**0.06**	0.02	1373	**0.09**	0.00	940
*Free chlorine (mg*/*L*)	**0.02**	0.32	2317	**−0.14**	0.00	1669
*Total chlorine (mg*/*L*)	**−0.08**	0.00	2317	**−0.36**	0.00	1669
*Chloride (mg Cl*^−^/*L*)	**n.a**	n.a	n.a	**0.41**	0.00	583
*Sulphate (mgSO*_*4*_^*2*−^/*L*)	**n.a**	n.a	n.a	**0.21**	0.00	294
*Conductivity (μS*/*cm at 20* °C)	**n.a**	n.a	n.a	**0.24**	0.00	925
*Fluoride (μg F*^−^/*L*)	**n.a**	n.a	n.a	**0.10**	0.08	293
*Ambient Temperature (°C)	**0.66**	0.00	24	**0.43**	0.04	24
*Rainfall (mm)	**0.18**	0.40	24	**0.33**	0.26	24

All parameters were measured according to modified in house analytical standard methods stablished by the United Kingdom Accreditation Service (UKAS) and collected from Jan. 2011 to Jan. 2013.

^*^Ambient temperature and rainfall correlations were performed using monthly average values taken from nine meteorological station (Data available online from http://www.metoffice.gov.uk/public/weather/climate-historic/).

**Table 2:** Quality variables data monitored at Scottish Water (Jan 2011–Jan 2013).

*Raw Water*	*Mean*	*Median*	*STDV*	*CV*	*1st Quartile*	*3rd Quartile*	*n*
*Alkalinity (mg HCO*_*3*_^−^/*L*)	**29.3**	20.0	29.3	1.0	10.0	45.0	411
*Ammonium (mg NH*_*4*_^*2*−^/*L*)	**0.03**	0.03	0.01	2.60	0.03	0.03	164
*Chloride (mg Cl*^−^/*L*)	**81.3**	104.5	50.8	1.6	24.3	119.7	16
*Colour (mg Pt*/*Co*/*L*)	**57.0**	42.0	51.2	1.1	23.0	76.0	2643
*Conductivity (μS*/*cm at 20* °C)	**80.3**	43.0	117.2	0.7	33.0	75.0	93
*pH*	**7.0**	7.2	0.6	11.9	6.7	7.4	2643
*DOC (mg C*/*L*)	**6.6**	5.4	5.2	1.3	3.3	8.3	1233
*Turbidity (NTU*)	**2.6**	1.0	14.0	0.2	0.5	1.8	2643
*UV Transmittance (%*)	**54.5**	58.4	15.6	3.5	46.5	65.2	88
*Bromide (μg*/*L*)	**64.1**	30.0	96.3	0.7	15.3	68.9	96
***Final Potable Water***							
*Alkalinity (mg HCO*_*3*_^−^/*L*)	**33.6**	25.0	32.3	1.0	16	41	795
*Ammonium (mg NH*_*4*_^+^/*L*)	**0.3**	0.2	0.1	3.3	0.2	0.3	1638
*Colour (mg Pt*/*Co*/*L*)	**3.2**	2.0	2.4	1.3	2	4	2850
*Conductivity (μS*/*cm at 20* °C)	**182.1**	199.0	69.3	2.6	136.8	222	512
*pH*	**7.9**	7.9	0.5	15.7	7.6	8.3	2986
*DOC (mg C*/*L*)	**1.8**	1.6	1.1	1.7	1	2.3	2402
*Turbidity (NTU*)	**0.3**	0.3	0.3	0.9	0.2	0.3	7759
*Bromodichloromethane (μg*/*L*)	**9.7**	7.1	8.4	1.2	3.9	13.5	2496
*Bromoform (μg*/*L*)	**2.6**	0.3	8.0	0.3	0.3	0.6	2497
*Chloroform (μg*/*L*)	**29.7**	21.3	27.4	1.1	8.8	44.1	2496
*Dibromochloromethane (μg*/*L*)	**6.2**	2.7	8.7	0.7	0.3	9.3	2497
*Total THM (μg*/*L*)	**48.0**	42.0	32.2	1.5	24.2	66.7	2493
*Chlorine free (mg*/*L*)	**0.5**	0.6	0.3	1.6	0.3	0.8	24901
*Chlorine total (mg*/*L*)	**0.9**	0.9	0.3	3.1	0.7	1.1	24892
***Distribution Networks***							
*Ammonium (mg NH*_*4*_^+^/*L*)	**0.1**	0.0	0.8	0.1	0.0	0.0	3088
*Chloride (mg*/*L*)	**17.8**	11.4	17.4	1.0	7.6	20.9	1079
*Colour (mg Pt*/*Co*/*L*)	**3.1**	2.0	6.1	0.5	2.0	3.0	3440
*Conductivity (μS*/*cm at 20* °C)	**125.7**	96.0	81.6	1.5	78.0	155.0	3423
*pH*	**8.0**	7.9	0.5	15.6	7.7	8.2	3463
*Sulphate (mg SO*_*4*_^*2*−^/*L*)	**17.1**	11.5	17.0	1.0	2.8	28.3	1079
*DOC (mg C*/*L*)	**1.7**	1.5	1.0	1.7	1.0	2.1	1809
*Turbidity (NTU*)	**0.3**	0.3	0.5	0.6	0.2	0.3	3449
*Bromodichloromethane (μg*/*L*)	**9.6**	7.1	8.5	1.1	3.9	12.1	1955
*Bromoform (μg*/*L*)	**2.0**	0.3	7.1	0.3	0.3	0.3	1958
*Chloroform (μg*/*L*)	**34.5**	25.9	31.3	1.1	11.2	50.8	1955
*Dibromochloromethane (μg*/*L*)	**4.8**	0.9	7.7	0.6	0.3	6.0	1959
*Total THM (μg*/*L*)	**50.5**	43.6	36.4	1.4	23.7	70.6	1954
*Chlorine free (mg*/*L*)	**0.3**	0.3	0.3	1.2	0.1	0.5	8781
*Chlorine total (mg*/*L*)	**0.6**	0.5	0.3	1.8	0.3	0.8	8780

STDV: Standard deviation; CV: Coefficient of Variation; n: number of entries. All parameters were measured according to modified in house analytical standard methods stablished by the United Kingdom Accreditation Service (UKAS).

**Table 3:** Total THMs and DOC per treatment type in Scottish Water sites.

Treatment	Additional Treatments	Dissolved Organic Carbon, mg C/L						DOC R. Efficiency%					Total trihalomethanes μg/L					
		Raw			Potable			Dist			Potable	Dist	Potable			Dist		
		Mean	*sd*	*n*	Mean	*sd*	*n*	Mean	*sd*	*n*	*%*	*%*	Mean	*sd*	*n*	Mean	*sd*	*n*
Coagulation	Dissolved Air Flotation_RGF	**6.8**	3.1	95	**1.66**	0.67	153	**1.47**	0.59	233	**75.6**	**78.4**	**41.2**	26.3	149	**38.1**	24.7	233
	Pressure Filtration	**11.2**	4.7	41	**1.84**	0.82	88	**1.82**	0.53	82	**83.6**	**83.7**	**39.1**	19.5	94	**39.9**	20.2	81
	Rapid Gravity Filtration	8.9	4.9	318	**2.12**	1.30	539	**1.94**	0.92	530	**76.2**	**78.2**	**44.8**	25.4	589	**56.2**	28.4	619
	Rapid Gravity Filtration_GAC	**4.8**	1.8	52	**1.87**	0.87	167	**2.06**	0.78	56	**61.1**	**57.1**	**64.9**	35.5	169	**83.3**	40.5	58
	Ultrafiltration	**4.6**	1.4	25	**1.58**	0.56	40	**1.55**	0.35	41	**65.6**	**66.3**	**21.1**	19.9	40	**16.0**	7.0	41
Membranes	Spiral_UF	**7.5**	6.7	104	**1.47**	0.59	337	**1.34**	0.58	145	**80.3**	**82.1**	**51.3**	26.4	334	**55.9**	34.4	154
	Multi tubular_NF	**5.8**	3.6	303	**1.40**	1.21	327	**1.05**	0.89	279	**75.9**	**81.9**	**46.1**	36.9	348	**36.9**	35.5	283
	Hollow fibre_UF	**2.3**	0.8	20	**1.95**	0.58	20	**2.26**	1.47	39	**15.2**	**1.9**	**18.0**	17.1	20	**25.6**	23.3	39
Unconventional	Coagulation & DynaSand	**7.2**	11	77	**2.13**	0.76	202	**1.83**	1.24	104	**70.4**	**74.5**	**45.0**	28.1	201	**54.4**	33.2	108
	Coagulation_Inverness Filter	**3.3**	3.8	42	**2.22**	1.05	153	**2.44**	1.21	61	**32.6**	**25.9**	**78.3**	34.1	159	**94.2**	36.3	67
	Ozone & GAC	**3.7**	0.8	77	**3.08**	0.67	59	**2.98**	0.69	26	**16.8**	**19.4**	**68.1**	20.4	69	**100.0**	21.2	58
No treatment	Disinfection only	**3.06**	3.2	79	**1.45**	1.11	288	**1.36**	1.17	177	**52.5**	**55.4**	**36.9**	37.5	291	**36.2**	47.5	179
	Disinfection_GAC	**1.8**	0.5	4	**1.59**	0.63	29	**1.53**	0.67	36	**11.5**	**15.1**	**35.4**	19.7	30	**38.9**	20.8	34

**UF:** Ultrafiltration (MWCO 8,000 Da).

**NF:** Nanofiltration (MWCO 2,000 Da).

**Inverness Filter**: Sand filtration unit with upward flow direction.

**DynaSand^®^** Sand filter backwash continuously.

**R. Efficiency%**: Removal Efficiency; RGF: Rapid Gravity Filtration; GAC: granulated activated carbon; Dist: Distribution networks; sd: standard deviation.

**Table 4:** Linear regression models for THMs in distribution networks during chlorination and chloramination (Jan. 2011–Jan. 2013).

Linear Regression models for trihalomethanes using Chlorination (*p* < 0.05)			r^2^	n
total THMs (μg/L)	=	52.0 + 1.4[T − 8.8](°C) + 0.4[Cl^−^ − 17.8](mg/L) + 26.9[DOC − 1.7](mg/L)	**0.76**	**502**
Chloroform (μg/L)	=	36.3 + 0.7[T − 8.8](°C) − 0.4[Cl^−^ − 17.8](mg/L) + 24.0[DOC − 1.7](mg/L)	**0.73**	**502**
Bromodichloromethane (μg/L)	=	10 + 0.2[T − 8.8](°C) + 0.3[Cl^−^ 17.8](mg/L) + 4.3[DOC − 1.7](mg/L)	**0.8**	**502**
Dibromochloromethane (μg/L)	=	3.3 + 0.06[T − 8.8](°C) + 0.3[Cl^−^ − 17.8](mg/L) − 0.4[DOC − 1.7](mg/L)	**0.84**	**502**
*Bromoform (μg/L)	=	18.3 + 1.1[T − 8.8](mg/L) + 1.2[Cl^−^ − 17.8](mg/L)	**0.66**	**144**
*Lowest limit of detection for bromoform (0.3 μg/L) eliminated for this model				
**Linear Regression models for trihalomethanes using Chloramination (*****p*** **<** **0.05)**			**r**^**2**^	**n**
Total THMs (μg/L)	=	13 −0.5[Cl^**−**^ − 17.8](mg/L) + 18.0[DOC − 1.7](mg/L)	**0.37**	**65**
Chloroform (μg/L)	=	8.2 − 0.9[Cl^−^ − 17.8](mg/L) + 13.7[DOC − 1.7](mg/L)	**0.38**	**65**
Bromodichloromethane (μg/L)	=	4.8 + 0.5[Cl^−^ − 17.8](mg/L) + 2.2[DOC − 1.7](mg/L)	**0.73**	**65**
Dibromochloromethane (μg/L)	=	N.A	—	—
Bromoform (μg/L)	=	N.A	—	—
