# Supplementary material for: Climatic, Geographic and Operational Determinants of Trihalomethanes (THMs) in Drinking Water Systems
Source: Sci Rep. 2016 Oct 20;6:35027. doi: 10.1038/srep35027 (PMC5071828; doi:10.1038/srep35027)
Supplement: Supplementary Information [file srep35027-s1.pdf]

Manuscript

CLIMATIC, GEOGRAPHIC AND OPERATIONAL DETERMINANTS OF TRIHALOMETHANES (THMS) IN DRINKING WATER SYSTEMS

Maria Valdivia-Garcia[†§], Paul Weir[§], Zoe Frogbrook[§], David W. Graham[†] and David Werner[†]*

[†] School of Civil Engineering and Geosciences

Newcastle University, Newcastle upon Tyne, United Kingdom

[§]Scottish Water, Castle House, Dunfermline, Edinburgh, United Kingdom

*To whom correspondence should be addressed.

E-mail: david.werner@ncl.ac.uk

Phone: (0044) 191 208 5099

Supplementary information

Regions in Scotland (strata)	Level	Group size, N_i	n_i = (N_i/2.9)
Clyde	1	6	2
Caithness	2	8	3
Orkney	3	11	4
Inverness	4	12	4
Ayr	5	13	4
Tay	5	13	4
Shetlands	5	16	6
North East Mainland	6	19	7
Nith	6	19	7
Western Isles	7	20	7
Fort William	8	21	7
Tweed	8	21	7
Skye & Lochalsh	8	21	7
West Coast	9	22	8
Forth	10	23	8
Argyll	11	25	9
**Total**	=	**270**	**93**

N_i : number of water treatment plants per region
n_i : selected water treatment plants per region

Table S1 Stratified sampling to determine the number of sites selected per region

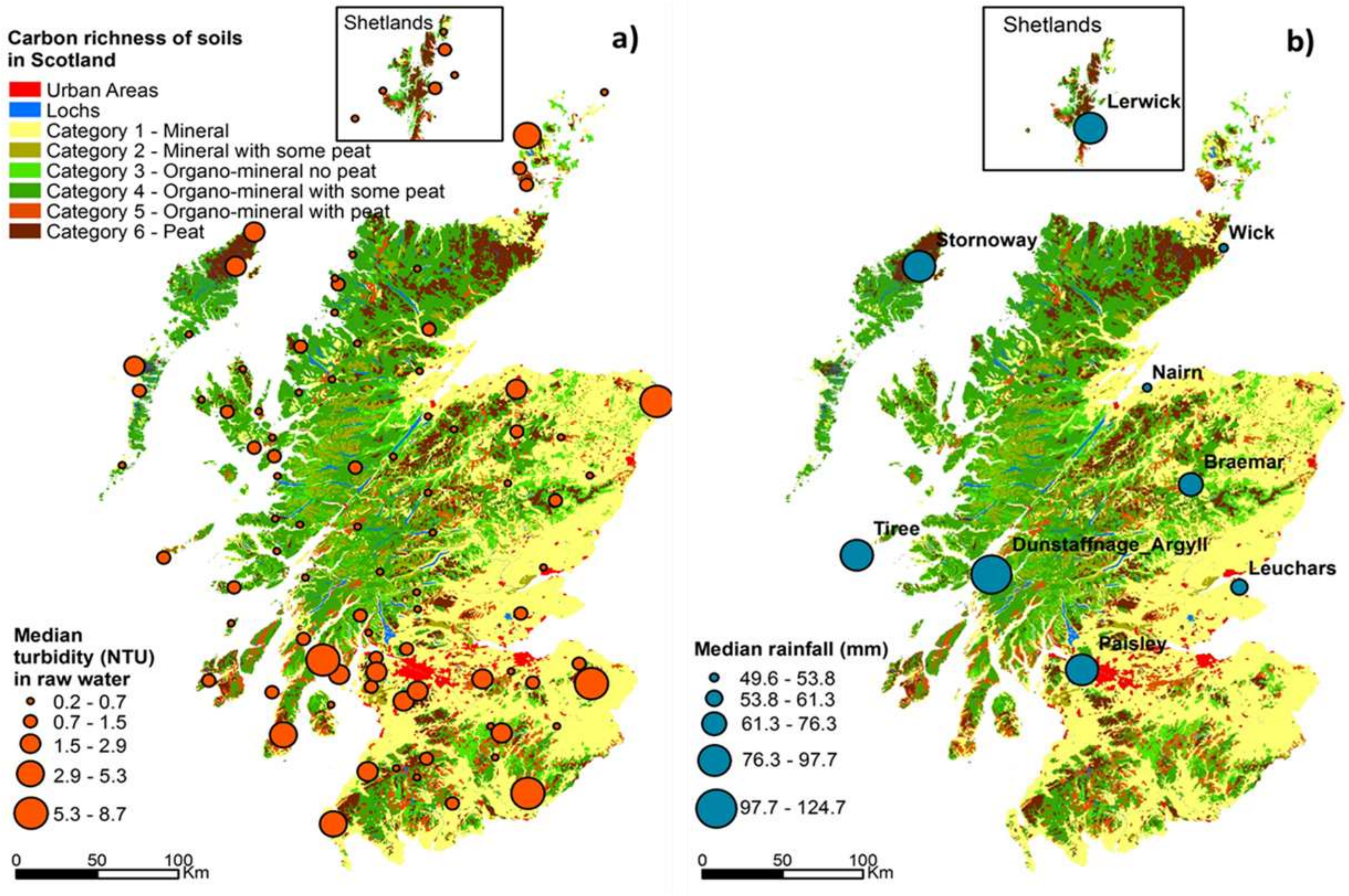

Figure S1 a) Spatial distribution of median turbidity (NTU) in raw water on soil types (Jan. 2011-Jan.2013) and b) median monthly rainfall values at nine meteorological stations around Scotland (Obtained using http://www.esri.com/news/arcnews/spring12articles/introducing-arcgis-101.html; version ArcMap 10.1)

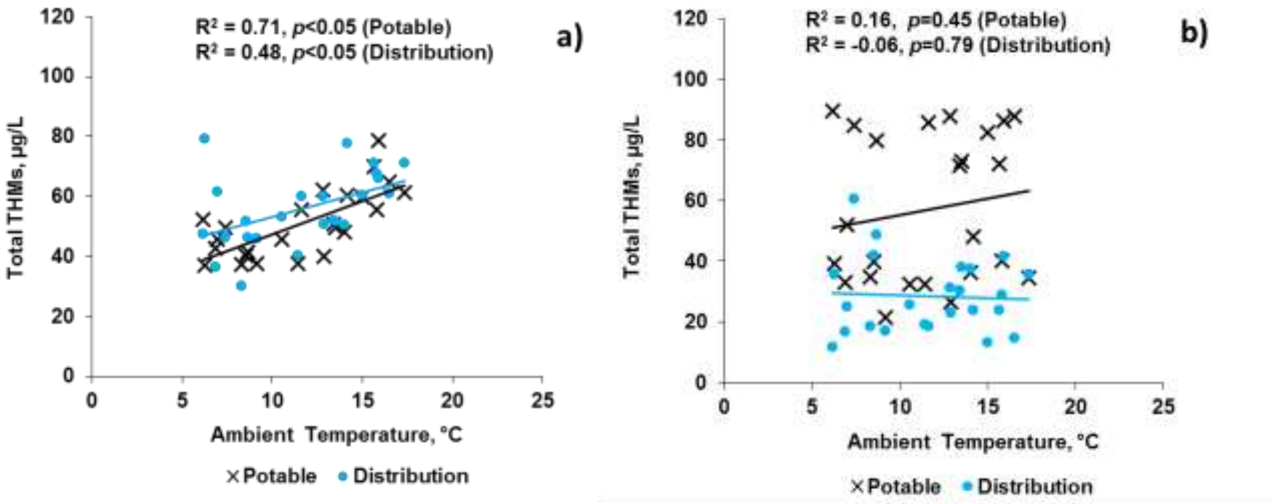

Figure S2 Correlations between monthly average total THMs concentrations and ambient temperature (Jan.2011-Jan.2013) in a) chlorination and b) chloramination WTPs (n = 24).
